# Certified Older Adult Peer Support Specialists' Use of Technology to Support Older Adults in the Community

**DOI:** 10.1093/geroni/igab046.493

**Published:** 2021-12-17

**Authors:** Karen Fortuna

**Affiliations:** Dartmouth College, Lebanon, New Hampshire, United States

## Abstract

Middle-aged and older adults with mental health conditions have a high likelihood of experiencing comorbid physical health conditions, premature nursing home admissions, and early death compared with the general population of middle-aged and older adults. An emerging workforce of certified older adult peer support specialists aged 50 years or above is one of the fastest growing mental health workforces and may be a suitable community-based workforce to simultaneously support the mental health, physical health, and aging needs of middle-aged and older adults with a serious mental illness. Older adult peer support specialists are people with a lived experience of aging into middle age and older adulthood with a mental health condition. This presentation will present three single-arm pilot studies examining how certified older adult peer support specialists’ incorporate technology, including text messaging, ecological momentary assessments, and smartphone applications into practice and clinical outcomes among older adults with serious mental illness.

